# Endosialin expression in soft tissue sarcoma as a potential marker of undifferentiated mesenchymal cells

**DOI:** 10.1038/bjc.2016.214

**Published:** 2016-07-19

**Authors:** Khin Thway, David Robertson, Robin L Jones, Joanna Selfe, Janet Shipley, Cyril Fisher, Clare M Isacke

**Affiliations:** 1 Sarcoma Unit, The Royal Marsden NHS Foundation Trust, 203 Fulham Road, London SW3 6JJ, UK; 2 The Breast Cancer Now Research Centre, The Institute of Cancer Research, 237 Fulham Road, London SW3 6JB, UK; 3 Sarcoma Molecular Pathology Team, Divisions of Molecular Pathology and Cancer Therapeutics, The Institute of Cancer Research, Sutton, Surrey SM2 5NG, UK

**Keywords:** endosialin, CD248, sarcoma, fibroblast, pericyte

## Abstract

**Background:**

Soft tissue sarcomas are a group of neoplasms with differentiation towards mesenchymal tissue, many of which are aggressive and chemotherapy resistant. Histology and immunoprofiles often overlap with neoplasms of other lineages, and establishing an accurate histopathological diagnosis is crucial for correct management, and therapeutic stratification. The endosialin cell surface glycoprotein is predominantly expressed by stromal fibroblasts and pericytes in epithelial neoplasms; however, tumour cell expression has been reported in small series of sarcomas.

**Methods:**

We assessed endosialin expression by immunohistochemistry in a large set of 514 human soft tissue sarcomas.

**Results:**

Tumour cell endosialin expression was seen in 89% of undifferentiated pleomorphic sarcomas (104/117), 77% adult fibrosarcomas/spindle cell sarcomas (20/26), 62% synovial sarcomas (37/60), 51% leiomyosarcomas (94/185) and 31% rhabdomyosarcomas (39/126).

**Conclusions:**

Endosialin immunohistochemistry has potential to distinguish undifferentiated and poorly differentiated sarcomas from other poorly differentiated, non-mesenchymal neoplasms. A Phase II trial randomising patients with advanced sarcomas to receive chemotherapy with/without an endosialin therapeutic antibody has recently completed enrolment. Endosialin expression could be used to select patients for such clinical trials. Based on our results, patients with undifferentiated pleomorphic sarcoma may be particularly suitable for such a therapeutic approach.

Soft tissue sarcomas are a complex group of childhood and adult neoplasms with differentiation towards mesenchymal tissue, which can arise almost anywhere in the body. While traditionally classified according to the mature mesenchymal tissue they most resemble, many subtypes are of uncertain differentiation with no normal cellular counterpart. As they present with similar clinical and radiologic findings, correct histopathological diagnosis is crucial, and morphology with ancillary immunohistochemistry remains the diagnostic cornerstone. This can be difficult in sarcomas lacking specific immunohistochemical differentiation and which overlap morphologically both with other sarcoma subtypes and with poorly differentiated non-mesenchymal neoplasms. Furthermore, many sarcomas behave aggressively, and surgery with or without radiation remains the mainstay of management for localised disease, as most, with the exception of select subgroups such as small round cell tumours, are resistant to conventional chemotherapy. As many sarcomas occur in sites where adequate surgical clearance is not possible, and the prognosis is generally poor for recurrent and metastatic disease, the need for novel rationally selected therapies is urgent. The discovery of more specific ancillary diagnostic immunohistochemical markers might aid the distinction of sarcomas from non-mesenchymal mimics, and enable better sub-classification into groups that will respond to specific targeted therapies.

Interest in endosialin (CD248) stemmed from the demonstration that it was the most highly upregulated transcript in colorectal cancer tumour vasculature, compared to the vasculature of normal adjacent tissue. This led to it being designated as ‘tumour endothelial marker 1' (St Croix *et al*, 2000). However, examination of a range of human cancers, although confirming this stromal upregulation of endosialin, has demonstrated that its expression is confined to stromal fibroblasts and pericytes of tumour vasculature rather than endothelium (MacFadyen *et al*, 2007; Rouleau *et al*, 2008; Simonavicius *et al*, 2008). Its expression and biologic role in sarcomas is little known. However, in comparison to other solid tumours, endosialin expression as detected by immunohistochemistry or mRNA analysis (Dolznig *et al*, 2005; Rouleau *et al*, 2008; O'Shannessy *et al*, 2016) has been reported in the tumour cell compartment in relatively small series of sarcomas. In view of this, the aim of this study was to assess the expression of endosialin in a large tissue microarray (TMA) of 514 human soft tissue sarcomas by immunohistochemistry. A second aim of this study was to obtain further data regarding endosialin expression in various sarcoma subtypes and stromal elements, in order to provide information for ongoing clinical trials of anti-endosialin agents.

## Materials and methods

All TMAs had multiple cores from each patient sample. The two rhabdomyosarcoma (RMS) TMAs comprised material (of both embryonal (ERMS) and alveolar (ARMS) subtypes) from clinical biopsies of patients (through the Children's Cancer and Leukemia Group, United Kingdom (Local Research Ethics Committee (LREC) protocol No. CCR-1836; Multi-Regional Research Ethics Committee/98/4/023 with consent where required) and Kiel Paediatric Tumour Registry, Department of Paediatric Pathology, University of Kiel, Germany respectively (Wachtel *et al*, 2006).

The adult soft tissue sarcoma TMAs were constructed as previously described (Kononen *et al*, 1998), and comprised cores from tumour biopsies of patients with synovial sarcoma, adult fibrosarcoma/spindle cell sarcoma not otherwise specified (i.e. for which all specific morphologic and genetic subtypes had been excluded), leiomyosarcoma and undifferentiated pleomorphic sarcoma (UPS) with appropriate approval for study (CCR-2015). Tumour diagnoses had been previously confirmed by a specialist soft tissue pathologist (CF). Synovial sarcomas comprised monophasic spindle cell neoplasms or biphasic spindle cell and epithelioid neoplasms with immunoprofiles and genetics as previously described (Thway and Fisher, 2014). Leiomyosarcomas comprised neoplasms with the appropriate morphology of intersecting fascicles of cells often with blunt-ended nuclei which expressed two or all of the muscle markers desmin, smooth muscle actin and h-caldesmon (typically uniformly but at least focally), and did not express markers of other lineages such as of skeletal muscle differentiation. Rhabdomyosarcomas all showed desmin expression, as well as nuclear expression of markers of skeletal muscle differentiation myogenin and MyoD1. The category ‘UPS' comprised morphologically pleomorphic soft tissue mesenchymal neoplasms in which no specific line of differentiation was identifiable morphologically or immunohistochemically. Tumours were only placed in this category when all possible lineages had been excluded (including dedifferentiated types of specific soft tissue sarcoma such as liposarcoma) (Fletcher *et al*, 2013).

Four-micrometre TMA sections were cut onto glass slides, dewaxed in xylene, then rehydrated through a series of ethanols into water. For antigen-retrieval slides were then pressure cooked for 2 min in 0.01 M pH 6.0 citrate target retrieval buffer, washed 5 min under running water and stained with the anti-endosialin monoclonal antibody B1/35.1 (MacFadyen *et al*, 2005), 1 mg ml^−1^ at a 1 : 500 dilution for 1 h at room temperature. Detection was achieved with the Vectastain avidin-biotin complex system according to the manufacturer's protocol (Vector Laboratories, Burlingame, CA, USA). Finally slides were dehydrated through a series of alcohols, cleared in xylene and mounted in 1,3-diethyl-8-phenylxanthine. Sections of normal human breast plus cores of normal human placenta within the TMAs served as positive immunohistochemical controls. Cores of liver and skeletal muscle served as negative controls.

Each core was first scored for staining intensity (absent, weak, moderate or strong) by a pathologist (KT), blinded to patient disease and outcome. Only well-defined, moderate or strong cell membranous and/or cytoplasmic staining was considered positive for this study, and ill-defined, nuclear or perinuclear staining was disregarded. Scoring was then performed both for tumour cells of each sarcoma and, when present in the cores, for tumour-associated stromal fibroblasts and pericytes. Immunoreactivity as shown in Table 1 was semiquantitatively evaluated as negative (i.e. 0% of cells stained), +1 (focally positive, 1–10% cells), +2 (positive in 11–50% of cells) or +3 (positive in >50% cells).

Following scoring, TMA slides were scanned using a Hamamatsu NanoZoomer-XR C12000, viewed in NDP.view2 and selected images exported into Adobe Photoshop CS6.

For confocal imaging, sections were subject to antigen retrieval as described above and then stained with anti-endosialin antibody B1/35.1 (2 μg ml^−1^) and anti-endomucin antibody (Abcam ab45771 (2 μg ml^−1^) or anti-*α*-smooth muscle actin (*α*SMA) antibody (Sigma-Aldrich, Dorset, UK; clone: 1A4, 0.4 μg ml^−1^), followed by AlexaFluor conjugated secondary antibodies (Molecular Probes, Eugene, OR, USA) as previously described (Simonavicius *et al*, 2008). Nuclei were counterstained with 14 nM DAPI (Molecular Probes), slides were rinsed in PBS and mounted with Vectashield (Vector Laboratories). Fluorescent images were collected sequentially in three channels on a Leica Microsystems TCS-SP2 confocal microscope. All images were taken at the same settings and exported from the Leica software into Adobe Photoshop CS6.

## Results

Tumour specimens from 514 patients were examined, and the findings for each sarcoma, summarised in Table 1, are described below:

### Controls

**Figure 1:**
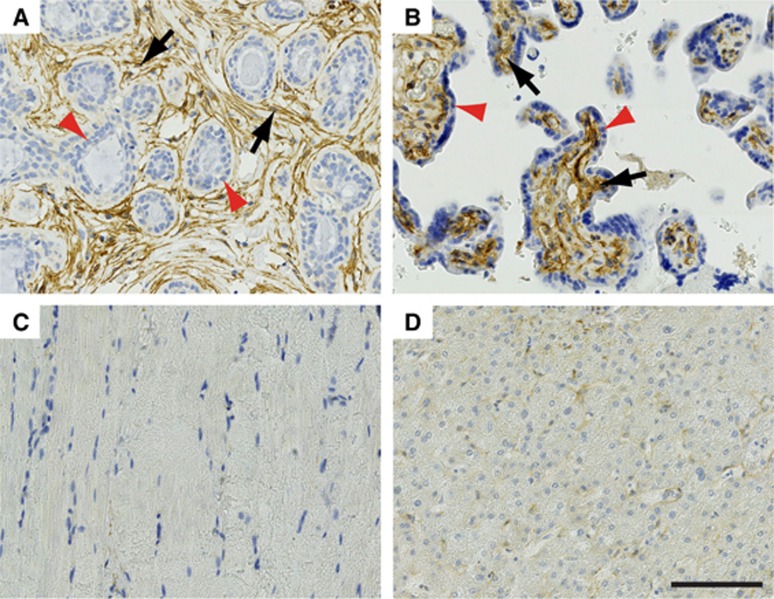
**Endosialin expression in control human tissues.** (**A**) Normal adult breast tissue shows strong endosialin expression on the stromal fibroblasts (black arrows) surrounding the endosialin-negative luminal and myoepithelial cells within the terminal duct lobular units (red arrowheads). (**B**) Placental tissue shows strong endosialin expression within the placental villi (black arrows), with absence of expression within the syncytiotrophoblastic cells (red arrowheads). (**C**, **D**) Normal skeletal muscle and liver tissue, respectively, show no endosialin expression. Scale bar, 100 *μ*m. See Supplementary Figure S1 for higher power images.

Figure 1 with higher power images shown in Supplementary Figure S1. Adult non-neoplastic human breast (positive control) showed, as previously reported (Simonavicius *et al*, 2008), strong endosialin expression on the stromal fibroblasts surrounding the terminal duct lobular units (Figure 1A). Normal human placenta (positive control) showed strong endosialin staining on the stromal cells within the placental villi surrounded by the endosialin-negative syncytiotrophoblastic cells (Figure 1B). Normal adult human skeletal muscle (Figure 1C) and liver (Figure 1D) control cores within TMAs were negative throughout.

### Undifferentiated pleomorphic sarcoma

**Figure 2:**
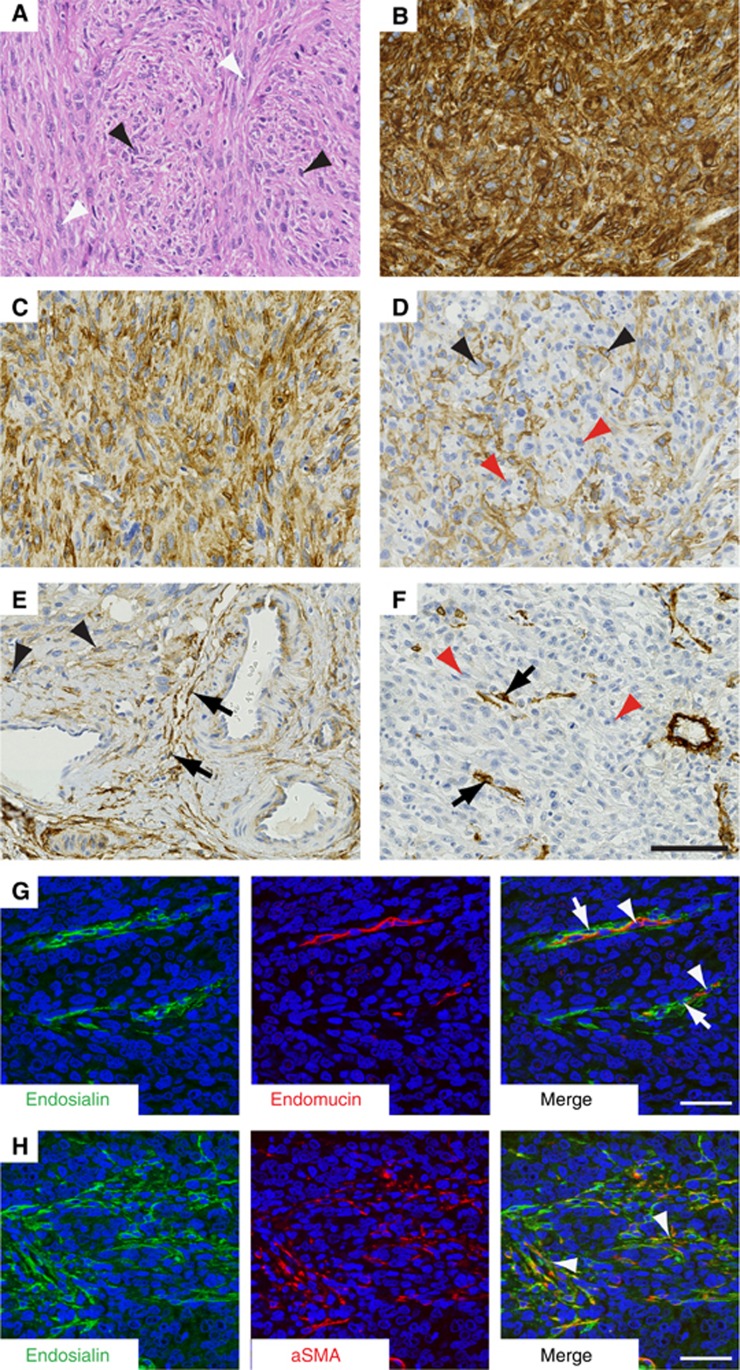
**Endosialin expression in undifferentiated pleomorphic sarcoma (UPS).** (**A**) Haematoxylin and eosin-stained section shows markedly pleomorphic, cellular tumour composed of sheets of spindle cells (black arrowheads) interspersed with ovoid cells (white arrowheads). (**B**, **C**) Examples 1 and 2. Both these UPS specimens show strong endosialin positivity, which is uniform throughout the tumour cells in panel **B**, but has a more focal, heterogeneous tumour cell distribution in panel **C**. (**D**) Example 3. UPS showing strong but focal membranous expression of endosialin (black arrowheads) on approximately 40% of tumour cells interspersed with endosialin-negative tumour cell (red arrowheads). (**E**) Example 4. The neoplastic cells (top left of field) show focal endosialin expression (black arrowheads), but endosialin expression is stronger within the surrounding stroma (black arrows). (**F**) Example 5. UPS with endosialin-negative tumour cells (red arrowheads), but endosialin is strongly expressed by the pericytes (black arrows). (**G**, **H**) Confocal microscopy of UPS specimens with stromal endosialin expression but no tumour cell positivity. Panel **G**, illustrating tumour vasculature with endosialin-positive pericytes (arrows) closely apposed to endomucin-positive endothelial cells (arrowheads). Panel **H** illustrating co-localisation of endosialin and *α*-smooth muscle actin in stromal fibroblasts (arrowheads). Scale bars, 100 *μ*m (panels **A**–**F**), 50 *μ*m (panels **G**, **H**). See Supplementary Figure S2 for higher power images.

Total of 117 patients (Figure 2A–H; higher power images shown in Supplementary Figure S2). Of the 104 UPS samples analysed, 104 (89%) had endosialin expression in tumour cells (Figure 2B–E; see Table 1 for full details). In addition, 94 (80%) displayed endosialin-positive stromal fibroblasts (see Figure 2E), and all 25 patients whose samples had detectable vasculature had endosialin-positive pericytes (see Figure 2F). To confirm expression of endosialin on stromal cells, UPS sections were co-stained for endosialin and either endomucin, as a marker of endothelial cells, or *α*-smooth muscle actin, as a marker of activated cancer-associated fibroblasts. As illustrated in Figure 2G, endosialin expression on the vasculature was restricted to pericytes, identified by their close apposition to endomucin-positive endothelial cells. As illustrated in Figure 2H, endosialin expression in the stromal bed was colocalised with *α*-smooth muscle actin-positive fibroblasts.

### Rhabdomyosarcoma

**Figure 3:**
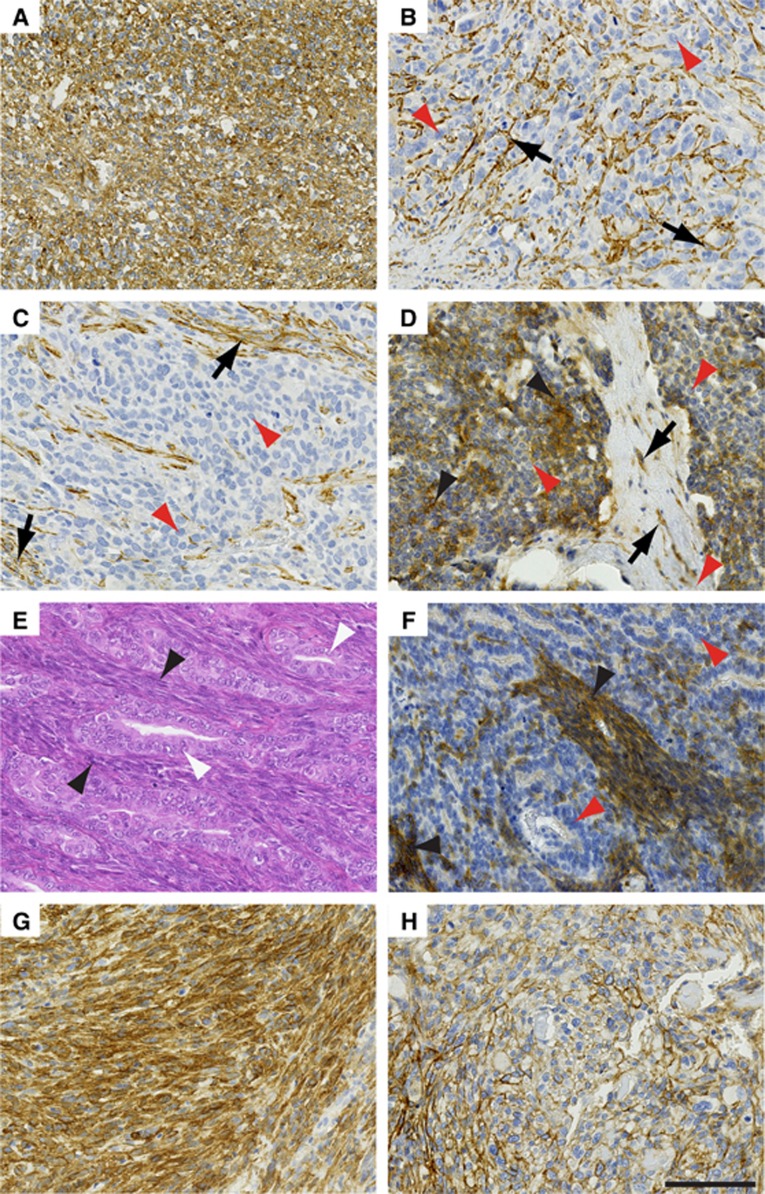
**Endosialin in expression in non-UPS sarcomas.** (**A**, **C**) Embryonal rhabdomyosarcoma. Panel **A**, Example 1 showing strong uniform expression of endosialin throughout the ovoid and spindle tumour cell populations. Panel **B**, Example 2 where the spindle and ovoid cells of the neoplasm show no expression of endosialin (red arrowheads), but strong endosialin expression on the pericytes of the tumour vasculature (black arrows). Panel **C**, Example 3–as in example 2 except additional expression of endosialin on stromal fibroblasts (black arrows). (**D**) Alveolar rhabdomyosarcoma showing focal expression of endosialin (black arrowheads) throughout the ovoid and spindle cell population, interspersed by endosialin-negative tumour cells (red arrowheads). Characteristic fibrous septa containing endosialin-positive fibroblasts (black arrows) divide the nests of round cells. (**E**, **F**) Biphasic synovial sarcoma. Panel **E**, haematoxylin and eosin-stained section showing spindle cell (black arrowheads) and glandular (white arrowheads) tumour cell components. Panel **F** illustrates an example with strong endosialin expression within the spindle cell component (black arrowheads), whereas the glandular component, composed of more rounded tumour cells, is largely endosialin-negative (red arrowheads). (**G**, **H**) Leiomyosarcoma. Panel **G** illustrates a tumour with high-level endosialin expression throughout. Panel **H** illustrates a tumour with focal endosialin expression. Scale bar, 100 *μ*m. See Supplementary Figure S3 for higher power images.

Total of 126 patients (Figure 3A–D; higher power images shown in Supplementary Figure S3A–D) comprising 100 embryonal rhabdomyosarcoma and 26 alveolar rhabdomyosarcoma, of which 35 (35%) (Figure 3A–C) and 4 (15%) (Figure 3D), respectively, contained endosialin expression in the tumour cells and 71 (71%) embryonal rhabdomyosarcoma and 12 (46%) alveolar rhabdomyosarcoma showed endosialin expression in stromal fibroblasts. Where the vasculature was evaluable (19 cases), all of the embryonal rhabdomyosarcoma and alveolar rhabdomyosarcoma tumours showed endosialin-positive pericytes.

### Synovial sarcoma

Total of 60 patients, of which 37 (62%) had endosialin expression in neoplastic cells (Figure 3E and F; Supplementary Figure S3E and F) and 46 (77%) showed expression in stromal fibroblasts (Figure 3F). Where the vasculature was evaluable (28 tumours), 100% had endosialin-positive pericytes associated with the vasculature.

### Adult fibrosarcoma/spindle cell sarcoma not otherwise specified

Total of 26 patients of which 20 (77%) had tumours expressing endosialin in tumour cells, 19 (73%) displayed expression of endosialin on stromal fibroblasts and all 5 tumours with evaluable vasculature had endosialin-positive pericytes.

### Leiomyosarcoma

Total of 185 patients (Figure 3G and H; Supplementary Figure 3G and H), of which 94 (51%) displayed endosialin expression in tumour cells, 107 (58%) had endosialin-positive stromal fibroblasts and 60/61 (98%) patients who had samples with detectable vasculature had endosialin-positive pericytes.

## Discussion

This study confirms that endosialin is expressed not only in pericytes and stromal fibroblasts of a range of human soft tissue sarcomas, but also in the neoplastic sarcoma cells. This is in contrast to the perivascular and fibroblastic distribution of endosialin expression previously reported in carcinomas and neoplasms of other lineages. Expression was seen in the neoplastic cells of 89% (*n*=104/117) of UPS, 77% of adult fibrosarcomas/spindle cell sarcomas (20/26), 62% of synovial sarcomas (37/60), 51% of leiomyosarcomas (94/185) and 31% of RMS (39/126).

Endosialin is a transmembrane glycoprotein encoded by the *CD248* gene in humans, and belonging to a family of C-type lectin transmembrane receptors. Its precise function remains to be understood, but it is associated with a role in angiogenesis during embryonic development, as well as postnatally in tumour development and growth and some inflammatory lesions (Rupp *et al*, 2006; Simonavicius *et al*, 2012). Endosialin is expressed by fibroblasts and pericytes in the embryo, and is downregulated during development, resulting in significant loss of expression in adult tissues (MacFadyen *et al*, 2007; Huang *et al*, 2011). In epithelial neoplasias, expression of endosialin is typically detected on stromal fibroblasts and tumour vessel-associated pericytes (MacFadyen *et al*, 2005; Rupp *et al*, 2006; MacFadyen *et al*, 2007; Bagley *et al*, 2008; Christian *et al*, 2008; Simonavicius *et al*, 2008) but not in the tumour cell compartment or endothelium (MacFadyen *et al*, 2005; Bagley *et al*, 2008; Christian *et al*, 2008; Simonavicius *et al*, 2008). This upregulated stromal expression of endosialin has been reported in a wide range of human cancers, including breast carcinomas (Rouleau *et al*, 2008), ovarian epithelial, colonic and rectal carcinomas (Bagley *et al*, 2008), small cell lung cancer, neuroblastoma and melanoma (Rouleau *et al*, 2011), metastatic melanomas and squamous cell carcinomas (Huber *et al*, 2006), high grade gliomas/glioblastoma multiforme, anaplastic astrocytomas and metastatic carcinomas to brain (Brady *et al*, 2004; Simonavicius *et al*, 2008). While its function in these stromal cells is yet to be clearly defined, knockout mouse models have shown the absence of endosialin expression results in reduced growth, invasion and metastasis of human tumour xenografts (Nanda *et al*, 2006; Tomkowicz *et al*, 2007), with increase in small immature vessels and decrease in medium and large tumour vessels, suggesting a role in controlling the interaction between tumour cells, endothelia and the extracellular matrix.

In contrast to the majority of human tumours of epithelial origin where endosialin expression is not detected on the tumour cells, tumour cell endosialin expression has been reported in a subset of neuroblastomas (Rouleau *et al*, 2011) and sarcomas. Regarding the latter, Rouleau *et al* assessed the immunohistochemical expression of endosialin on 86 formalin-fixed, paraffin-embedded human clinical sarcoma specimens (Rouleau *et al*, 2008), and documented expression in different cell types; 51% (54/86) showed endosialin expression in malignant cells, 78% (67/86) in vasculature and 22% (19/86) in stromal cells. Endosialin expression was also found in 22/42 human sarcoma cell lines screened *in vitro*, with a positive correlation between mRNA and protein levels. When implanted *in vivo*, expression was seen at all sites of tumour dissemination. Recent studies by this group on endosialin-positive sarcoma cell lines have shown maintenance of endosialin expression in sarcoma side population cells with stem-cell like properties, reinforcing the hypothesis that endosialin is a potential therapeutic target in sarcoma (Rouleau *et al*, 2012; Sun *et al*, 2015).

Using a larger sample of human clinical tumour specimens from 514 patients, we have demonstrated endosialin expression in some of the most common sarcoma subtypes. Consistent with our data, Rouleau *et al* (2008) reported endosialin protein expression in sarcoma cells in 80% (8/10) of synovial sarcomas, 75% (6/8) of fibrosarcomas, 73% (8/11) of UPS and 10% (1/10) of RMS, with similar results obtained in an independent series (O'Shannessy *et al*, 2016). The high expression of endosialin shown in soft tissue sarcomas (rather than only in tumour stroma and pericytes) suggests it might have potential diagnostic utility in discriminating sarcomas from other neoplasms. A significant diagnostic hurdle in soft tissue pathology is the lack of a reliable immunohistochemical marker of mesenchymal lineage. While vimentin, a type III intermediate filament protein forming the major cytoskeletal component of mesenchymal cells, is commonly used in diagnostic immunohistochemical panels, it is also expressed extensively in neoplasms of other lineages, making it an ineffective diagnostic marker.

The high expression of endosialin seen in UPS in our study suggests that its expression might be upregulated in sarcomas as a consequence of its undifferentiated phenotype. Endosialin expression was also present in almost 80% of fibrosarcomas/ spindle cell sarcomas not otherwise specified in this study; these are a group of spindle cell neoplasms with fascicular or ‘herringbone' architecture and which also lack a specific immunophenotype, again supporting the possibility that endosialin might be an indicator of both ‘mesenchymal' differentiation and lack of differentiation. Endosialin is expressed in a smaller proportion of synovial sarcomas, leiomyosarcomas and RMS, which are each indisputably better differentiated, having distinct morphologic patterns and immunoprofiles.

In sarcomas endosialin is expressed in both neoplastic cells and those of tumour stroma, as demonstrated by our co-immunofluorescent staining. This could have importance in the interaction between tumour cells, tumour fibroblasts and pericytes leading to neoplastic progression and dissemination. Ontuxizumab (MORAb-004) is a humanised monoclonal antibody targeting endosialin, and has shown preliminary anti-tumour activity in a Phase I trial in treatment-refractory solid tumours, including sarcoma patients (Diaz *et al*, 2015). Our results are relevant, as a randomised Phase II trial of ontuxizumab has recently completed enrolment. Patients with advanced soft tissue sarcoma were randomised to receive gemcitabine/ docetaxel with the antibody or with placebo. Patients were stratified into four groups: liposarcoma, UPS, leiomyosarcoma and a heterogeneous group of other subtypes. Consequently, further data on endosialin expression in individual subtypes, particularly UPS, are very important and may guide further evaluation of this antibody.

While further work is required in assessing endosialin expression in other sarcoma types, particularly to further explore the differential expression of endosialin on differentiated *vs* less differentiated sarcomas, this study suggests that endosialin may be of potential use as an ancillary diagnostic aid in three ways: first, as a putative marker of ‘mesenchymal' lineage, second in discriminating pleomorphic sarcomas from poorly differentiated non-mesenchymal neoplasms and finally as a potential marker of the progressive ‘undifferentiation' of mesenchymal tumours.

## Figures and Tables

**Table 1:** Immunohistochemical expression of endosialin in soft tissue sarcoma tissue microarrays

		**Tumour cell expression**				**Stromal fibroblast expression**				**Pericyte expression**			
**Sarcoma type**	**No. of patients**	**+1**	**+2**	**+3**	**Total positive**	**+1**	**+2**	**+3**	**Total positive**	**+1**	**+2**	**+3**	**Total positive**
Undifferentiated pleomorphic sarcoma (UPS)	117	8	21	75	104/117 (88.9%)	11	16	67	94/117 (80.3%)	2/25	11/25	12/25	25/25 (100%)
Embryonal rhabdomyosarcoma (ERMS)	100	13	14	8	35/100 (35.0%)	25	25	21	71/100 (71.0%)	6/78	29/78	43/78	78/78 (100%)
Alveolar rhabdomyosarcoma (ARMS)	26	2	1	1	4/26 (15.4%)	4	3	5	12/26 (46.2%)	2/19	2/19	15/19	19/19 (100%)
Synovial sarcoma	60	14	14	9	37/60 (61.7%)	12	20	14	46/60 (76.7%)	2/28	13/28	13/28	28/28 (100%)
Adult fibrosarcoma/spindle cell sarcoma of no special type	26	6	8	6	20/26 (76.9%)	2	9	8	19/26 (73.1%)	0/5	3/5	2/5	5/5 (100%)
Leiomyosarcoma	185	16	31	47	94/185 (50.8%)	22	37	48	107/185 (57.8%)	7/61	26/61	27/61	60/61 (98.4%)
