# Supplementary material for: Endosialin expression in soft tissue sarcoma as a potential marker of undifferentiated mesenchymal cells
Source: Br J Cancer. 2016 Jul 19;115(4):473–9. doi: 10.1038/bjc.2016.214 (PMC4985356; doi:10.1038/bjc.2016.214)
Supplement: Supplementary Information [file bjc2016214x1.pdf]

Endosialin expression in soft tissue sarcoma as a potential marker of undifferentiated mesenchymal cells

Khin Thway, David Robertson, Robin L. Jones, Joanna Selfe, Janet Shipley, Cyril Fisher and Clare M. Isacke

Supplementary Material

Supplementary Figure S1
Supplementary Figure S2
Supplementary Figure S3

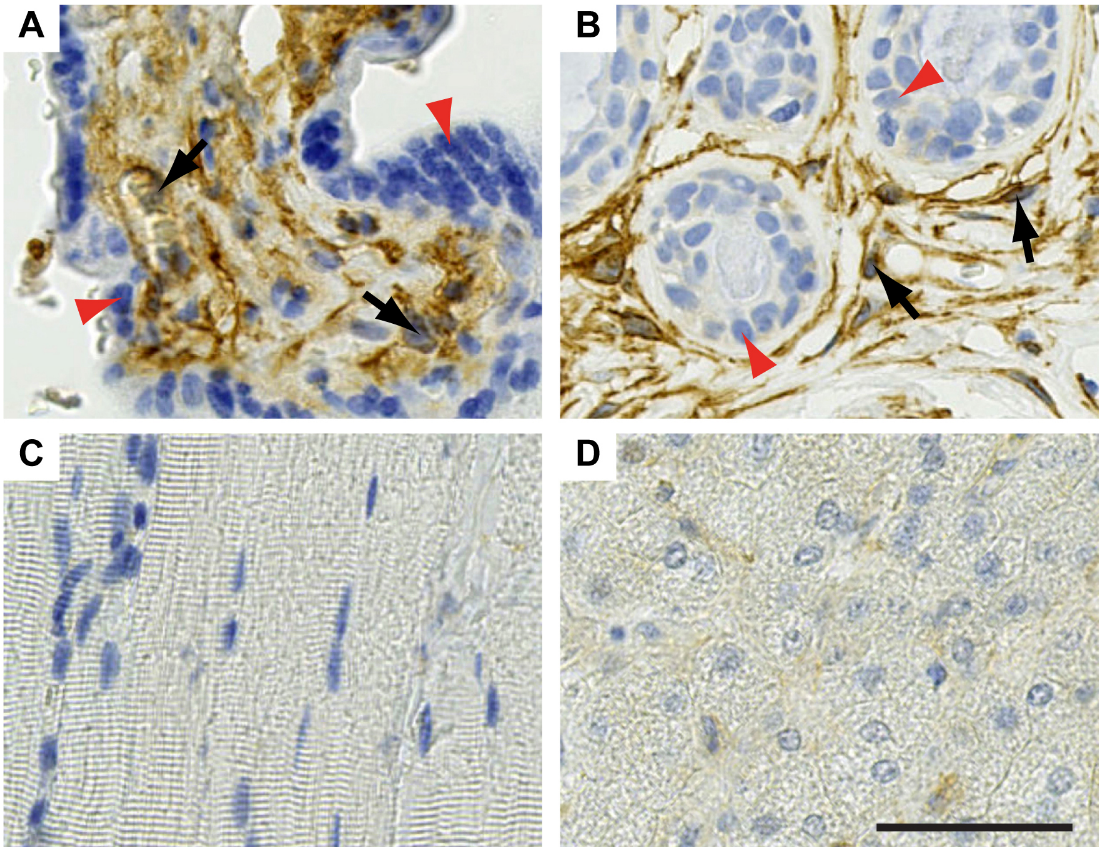

Supplementary Figure S1. Endosialin expression in control human tissues.

Higher power images of panels shown in Figure 1. **(A)** Placenta. Black arrowheads endosialin-positive stromal cells within the placental villi. Red arrowheads, endosialin-negative syncytiotrophoblastic cells. **(B)** Normal adult breast. Black arrowheads, endosialin-positive stromal fibroblasts. Red arrowheads, endosialin-negative luminal and myoepithelial cells. **(C)** Skeletal muscle, **(D)** Liver. Scale bar, 50 μm.

Supplementary Figure S2

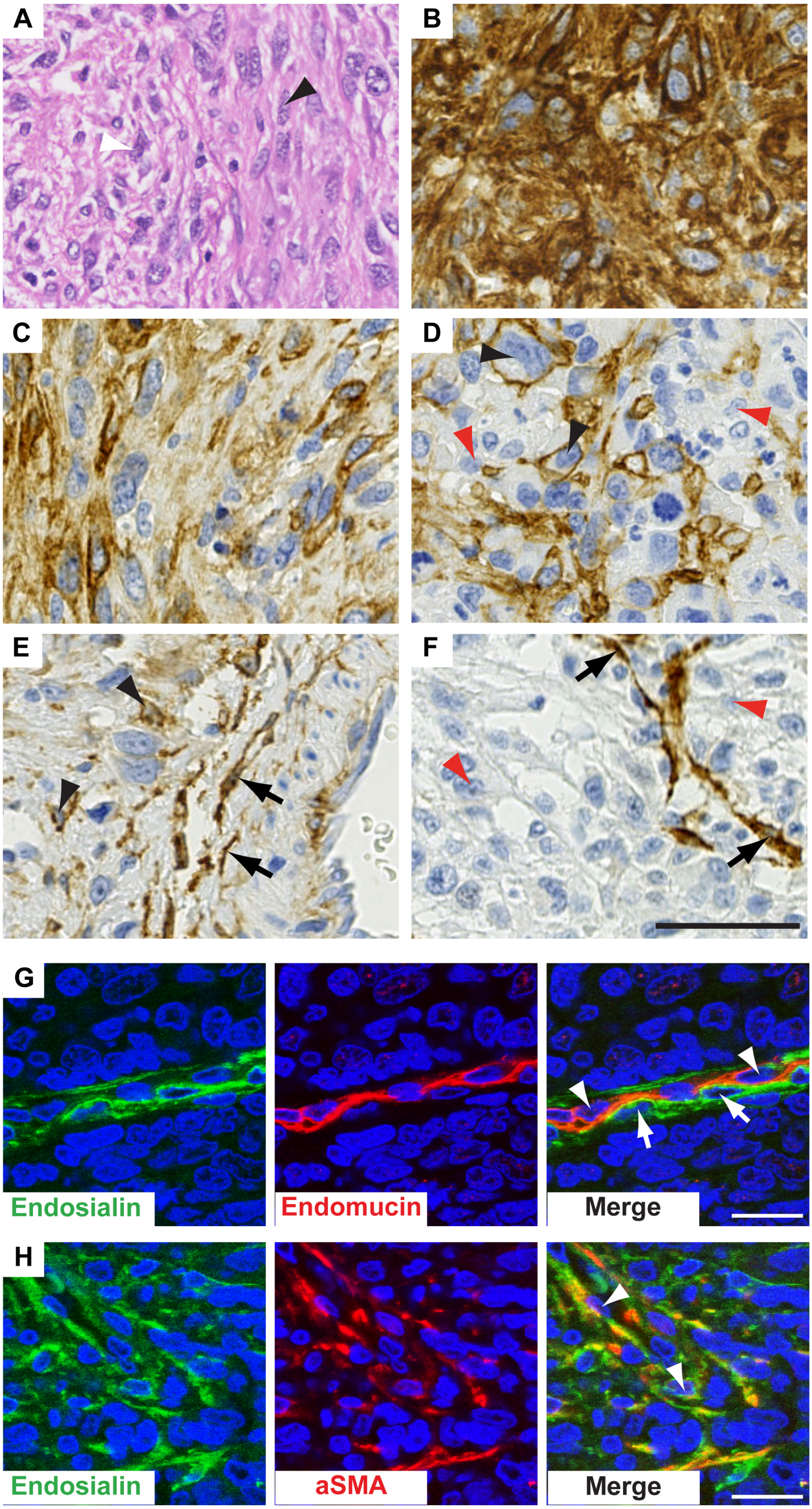

Figure 2. Endosialin expression in undifferentiated pleomorphic sarcoma (UPS).

Higher power images of panels shown in Figure 2. **(A)** Haematoxylin and eosin stained UPS section illustrating sheets of spindle tumour cells (black arrowhead) interspersed with ovoid tumour cells (white arrowheads). **(B,C)** Two UPS specimens showing strong endosialin positivity within the tumour cells, which is uniform in panel B example, but more focal in panel C example. **(D)** UPS showing strong but focal tumour cell endosialin expression (black arrowheads) interspersed with endosialin-negative tumours cells (red arrowheads). (**E**) Neoplastic cells (top left of field) show focal endosialin expression (arrowheads), but endosialin expression is stronger within the surrounding stroma (arrows). (**F**) UPS with endosialin-negative tumour cells (red arrowheads) but strong endosialin expression by the pericytes (arrows). (**G,H**) Examples of UPS with stromal endosialin expression but no tumour cell positivity. Panel G, illustrating tumour vasculature with endosialin-positive pericytes (arrows) closely apposed to endomucin-positive endothelial cells (arrowheads). Panel H illustrating co-localisation of endosialin and αSMA in stromal fibroblasts (arrowheads). Scale bars, 50 µm (panels A-F), 25 µm (panels G,H).

Supplementary Figure S3

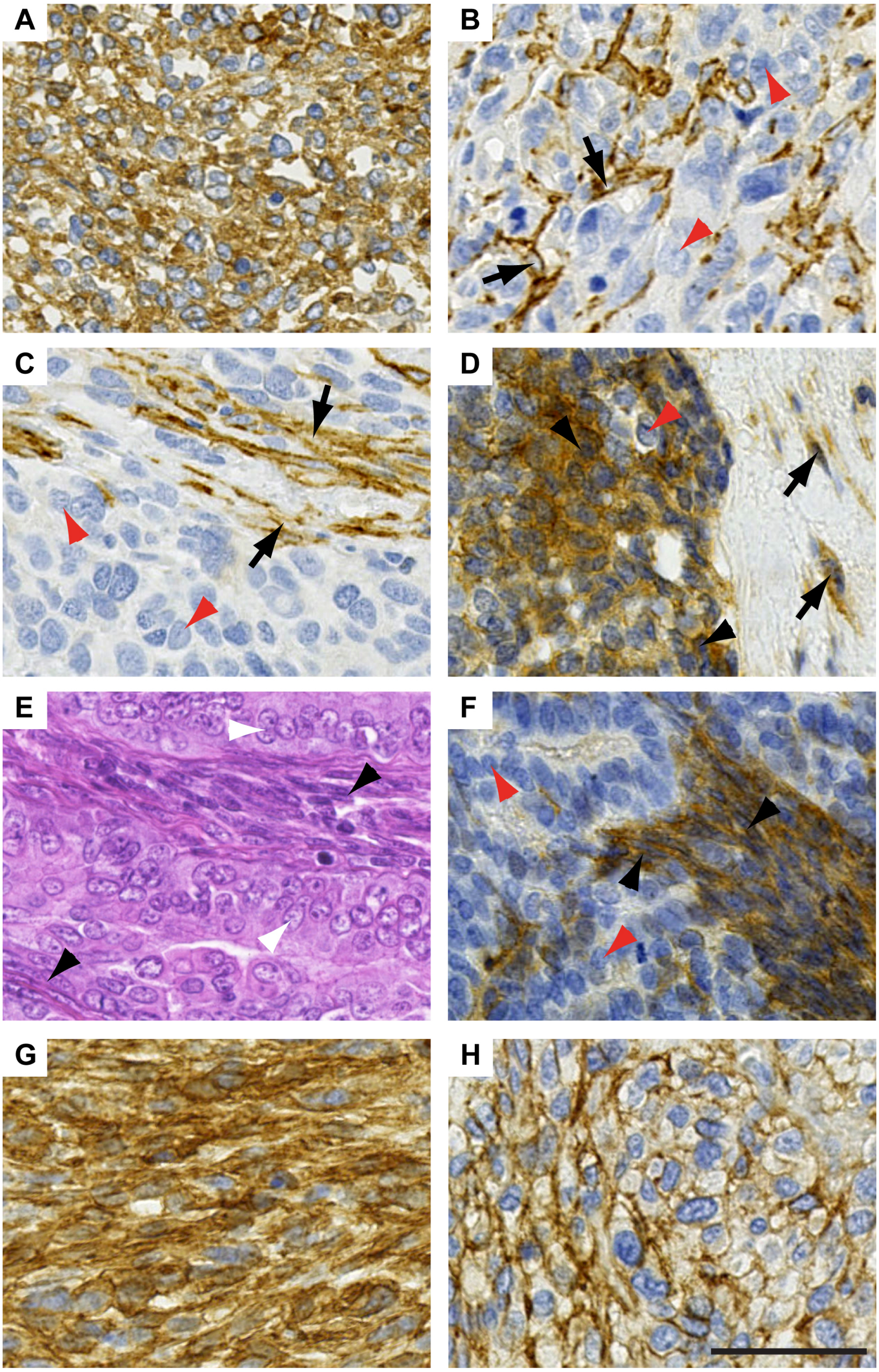

Figure 3. Endosialin in expression in non-UPS sarcomas

Higher power images of panels shown in Figure 3. **(A,C)** Embryonal rhabdomyosarcoma (ERMS). (**A**) ERMS with strong expression of endosialin throughout the ovoid and spindle tumour cell populations. (**B**) ERMS with endosialin-negative tumour cells (red arrowheads), but strong endosialin expression on the

pericytes (arrows). (**C**) ERMS with endosialin-negative tumour cells (red arrowheads), but strong endosialin expression on the stromal fibroblasts (arrows). (**D**) Alveolar rhabdomyosarcoma (ARMS) showing focal endosialin expression within the sheets of round cells (black arrowheads), interspersed with endosialin-negative tumour cells (red arrowheads). Characteristic fibrous septa dividing the nests of round cells contain endosialin-positive fibroblasts (arrows). **(E,F**) Biphasic synovial sarcoma. Panel E, H&E-stained section showing spindle cell (black arrowheads) and glandular (white arrowheads) tumour cell components. Panel F illustrates an example with strong endosialin expression within the spindle cell component (black arrowheads) surrounded by endosialin-negative rounded cells of the glandular component (red arrowheads). (**G,H**) Leiomyosarcoma. Panel G illustrates a tumour with high-level endosialin expression throughout. Panel H illustrates a tumour with focal endosialin expression. Scale bar, 50 μm.
